# Calcite Kinks Grow via a Multistep Mechanism

**DOI:** 10.1021/acs.jpcc.2c04116

**Published:** 2022-09-13

**Authors:** Alexander Broad, Robert Darkins, Dorothy M. Duffy, Ian J. Ford

**Affiliations:** London Centre for Nanotechnology, University College London, 17-19 Gordon Street, London WC1H 0AH, U.K.

## Abstract

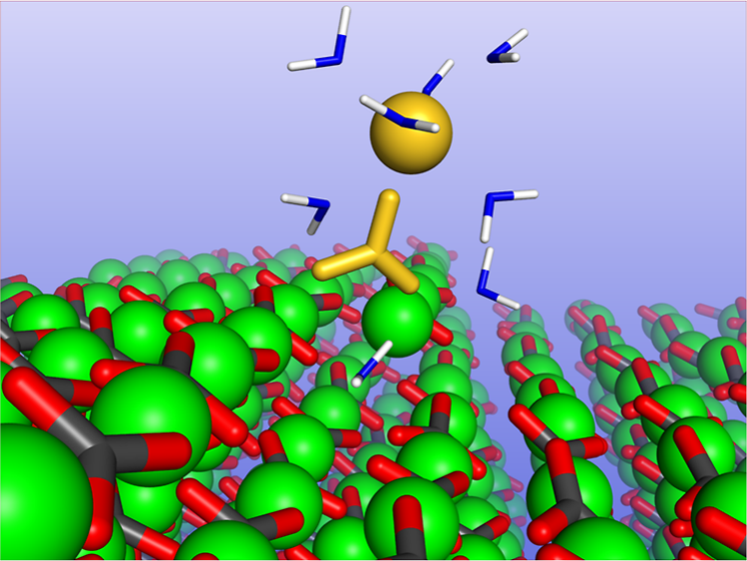

The classical model of crystal growth assumes that kinks grow via a sequence of independent adsorption events where each solute transitions from the solution directly to the crystal lattice site. Here, we challenge this view by showing that some calcite kinks grow via a multistep mechanism where the solute adsorbs to an intermediate site and only transitions to the lattice site upon the adsorption of a second solute. We compute the free energy curves for Ca and CO_3_ ions adsorbing to a large selection of kink types, and we identify kinks terminated both by Ca ions and by CO_3_ ions that grow in this multistep way.

## 1 Introduction

Calcite, a common biomineral and the most abundant carbonate on Earth, has been the subject of extensive experimental and computational study. However, our understanding of the molecular mechanisms underpinning calcite growth remains limited. While ion adsorption to calcite steps has been comprehensively studied using molecular simulation,^1^ studies of kinks have been limited to only a few kink types^2,3^ or focused on energy calculations on non-aqueous calcite.^4^ Calcite kink growth is a complex process involving the attachment and detachment of ions to and from 16 unique kink sites. A more comprehensive study is therefore required to gain insights into the molecular processes that constitute calcite kink growth.

Crystal growth models usually treat the attachment of units to kinks as independent elementary events, where the kinks are structured such that the terminating ion occupies its lattice site upon adsorption.^5−12^ The structure of kink sites has consequences for the study of the interactions between impurities and kinks. The role of impurities in crystallization has been the focus of many experimental studies, ranging from their retardation of crystal growth^13−15^ to their morphological and mechanical impact.^16,17^ Molecular simulation is able to complement such studies through determining binding configurations^18^ and calculating binding free energies.^15,19−21^ However, such studies assume that the terminating ions of kinks occupy their lattice site. Computational studies of kink nucleation have already revealed a more complex picture where lone CO_3_ ions are found to prefer to reside above the step, rather than adsorbing directly to the lattice sites.^1^ A similar complex process has been observed for barite.^22^ Because kinks do not nucleate through the straightforward process of direct adsorption of units onto crystal lattice sites, we cannot assume that they propagate in such a way.

In this study, we determine the free energy curves for Ca and CO_3_ ions adsorbing to 12 out of 16 kink types, revealing each thermodynamically stable kink configurations. By simulating the dual adsorption of a CO_3_ ion and a Ca ion to a kink, we show that ions that adsorb preferentially to a bidentate configuration will transition to the lattice site after a second ion adsorbs.

## 2 Methodology

**Figure 1:**
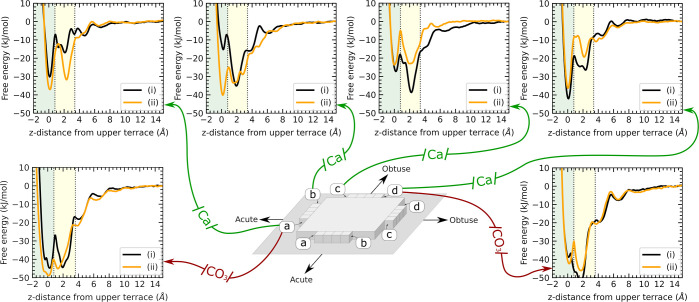
Free energy profiles for Ca and CO_3_ ions adsorbing to various kink types. Letters a–d denote the kink geometry (as shown in the schematic) and the labels (i) and (ii) represent the distinct kinks due to the alternating CO_3_ orientations along each step. A value of zero along the *x*-axis corresponds to the adsorbate residing along the same {10.4} plane as the upper terrace, i.e., residing in the lattice site of the kink. The highlighted green and yellow regions correspond to the lattice and bidentate configurations (see Figure 2).

The free energy surfaces for Ca and CO_3_ ions adsorbing to a selection of calcite kinks were computed using metadynamics. Calcite has 16 unique kinks on each side of its glide plane: four kink geometries, labeled (a–d) in Figure 1, each of which can be terminated by either Ca(i), CO_3_(i), Ca(ii), or CO_3_(ii), where (i) and (ii) represent the alternating carbonate orientations along each step. The free energy surfaces were computed for a Ca ion adsorbing to each of the eight CO_3_-terminated kinks; a CO_3_ ion adsorbing to four of the eight Ca-terminated kinks; and the dual adsorption of a CO_3_ ion and a Ca ion to one type of Ca-terminated kink. For each kink site, the ion that terminates the kink is tethered to the exact position of its bulk lattice site using a radial harmonic potential with a spring constant of 100 kJ/mol/Å. When this was applied to CO_3_, only the C atom was constrained in this potential. Additionally, in order to prevent its drift, the calcite slab was held in place by setting the total momentum of the slab (excluding the adsorbate) to zero at every timestep.

Molecular dynamics simulations were performed using LAMMPS.^23^ The inter- and intra-molecular interactions for Ca and CO_3_, as well as their interactions with water, were described by the force field of Raiteri et al.^24^ which was explicitly fitted to reproduce the experimentally found solubility of calcite. The self-interactions of water were described by SPC/Fw.^25^ Periodic boundaries were used in all dimensions, and a monoclinic skew was added to the simulation box in order to accommodate a slab of calcite periodic in the *x*- and *y*-directions, including an elevated step and two exposed kink sites. The slab of calcite was separated from its periodic image in the *z*-direction by a gap filled with water molecules. Free energy surfaces were computed using well-tempered metadynamics as implemented in Plumed.^26^ The reaction coordinates depended on the simulation, as summarized below. The results we present in this paper show the free energy as a function of the distance normal to the {10.4} plane between the adsorbate (or the C atom in the CO_3_ case) and the step. Further simulation details can be found in the Supporting Information.

The simulation free energy differences, which we denote with Δ*G*_sim_, can be extracted from the free energy surfaces by determining the difference between the minimum free energy, and the free energy of the fully dissolved solute that is where the free energy surface becomes flat. In this paper, we calculate the simulation free energies by averaging the free energy surfaces between 15 and 18 Å above the upper terrace. Figure S4 in the Supporting Information details the region over which the free energies are averaged to calculate Δ*G*_sim_. The adsorption free energy, which we denote with Δ*G*_ads_, is a reference free energy given by
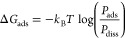
1where *P*_ads_ and *P*_diss_ are the equilibrium probabilities of finding a solute in an adsorbed and dissolved state, respectively, when the solute is dissolved in solution at a concentration of 1 mol. As the adsorption free energy has a well-defined meaning and does not depend on any experimental or simulation conditions, it makes an ideal quantity to calculate. Δ*G*_ads_ can be computed with an entropic correction to Δ*G*_sim_ as detailed in the Supporting Information.

### 2.1 Ca Adsorption

For a Ca ion adsorbing to a CO_3_-terminated kink, the *z* coordinate of the Ca ion [its position normal to the (10.4) surface] was chosen as the reaction coordinate. The Ca ion was confined to a region in the (*x*, *y*) plane, centered on the target adsorption site using harmonic barriers. Explicitly dehydrating the Ca ion can be important in some reactions,^1^ but it had negligible effect on the free energy surfaces in the case of Ca adsorption to kinks (see the Supporting Information).

The average number of water molecules coordinated with the adsorbing Ca ion, ⟨*N*_c_⟩, was measured while the ion was in its most stable bound configuration
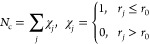
2where *r*_*j*_ is the distance to water oxygen *j* and *r*_0_ = 3 Å is a cutoff distance. The value of 3 Å is chosen from radial distribution functions previously computed by Raiteri et al.^24^

### 2.2 CO_3_ Adsorption

For a CO_3_ ion adsorbing to a Ca-terminated kink, the CO_3_ ion was confined to a region in the (*x*, *y*) plane using harmonic barriers, and two reaction coordinates were used: the *z* coordinate of the CO_3_ ion and, to drive dehydration of the kink, the distance between the kink-terminating Ca ion and its nearest water molecule. More precisely, we used an approximation of this nearest distance
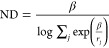
3where *r*_*j*_ is the distance to water oxygen *j* and β is a constant. For an appropriately chosen β, this function is a continuously differentiable approximation of the smallest *r*_*j*_, making it suitable as a reaction coordinate (a similar approximation has been used elsewhere^27^). A justification for this reaction coordinate can be found in the Supporting Information.

### 2.3 Dual CO_3_ and Ca Adsorption

Starting with the *d*(i) Ca-terminated kink, the adsorption of a CO_3_ ion and the subsequent Ca ion was simulated using three reaction coordinates: the hydration state of the kink-terminating Ca ion as characterized by ND (eq 3), and the *z* coordinates of the two adsorbates. Each adsorbate was confined with its own set of harmonic potentials, each constraining the Ca or C atom to within 2 Å of its respective lattice site in the *x*- and *y*-direction (as per the method outlined in Section 1.4 of the Supporting Information). The adsorbing CO_3_ ion was constrained to a *z* value less than 4 Å above the kink site.

## 3 Results and Discussion

### 3.1 Ca Adsorption Free Energies

We calculated the free energy surfaces for a Ca ion adsorbing to eight different CO_3_-terminated kinks as a function of the Ca-kink *z*-distance (Figure 1). Table 1 summarizes the simulation free energy differences and corresponding adsorption free energies, as well as the average number of water molecules coordinated with the Ca ion in its most stable bound configuration, ⟨*N*_c_⟩.

**Table 1:** Simulation Free Energy Differences (Δ*G*_sim_) and Adsorption Free Energies (Δ*G*_ads_) for Ca and CO_3_ Ions Adsorbing to Various Kink Types. ⟨*N*_c_⟩ is the average water coordination number of the Ca adsorbate in its most stable configuration.

ion	kink	Δ*G*_sim_ (kJ/mol)	Δ*G*_ads_ (kJ/mol)	⟨*N*_c_⟩
Ca	*a*(i)	–30	–20	3.3
	*a*(ii)	–36	–26	3.2
	*b*(i)^a^	–35	–25	4.6
	*b*(ii)	–31	–20	3.2
	*c*(i)^a^	–39	–29	4.5
	*c*(ii)	–24	–15	3.1
	*d*(i)	–42	–31	2.8
	*d*(ii)	–36	–26	2.7
CO_3_	*a*(i)^a^	–46	–32	-
	*a*(ii)	–49	–36	-
	*d*(i)^a^	–52	–38	-
	*d*(ii)^a^	–46	–33	-

a The ion adsorbs preferentially to the bidentate configuration.

The Δ*G*_ads_ values shown in Table 1 vary between −14.7 and −30.8 kJ/mol. The variation of these numbers is unsurprising because similar calculations for step sites show significant variation in binding free energies.^1^ Nevertheless, it is clear that different Ca-terminated kink sites have different stabilities.

**Figure 2:**
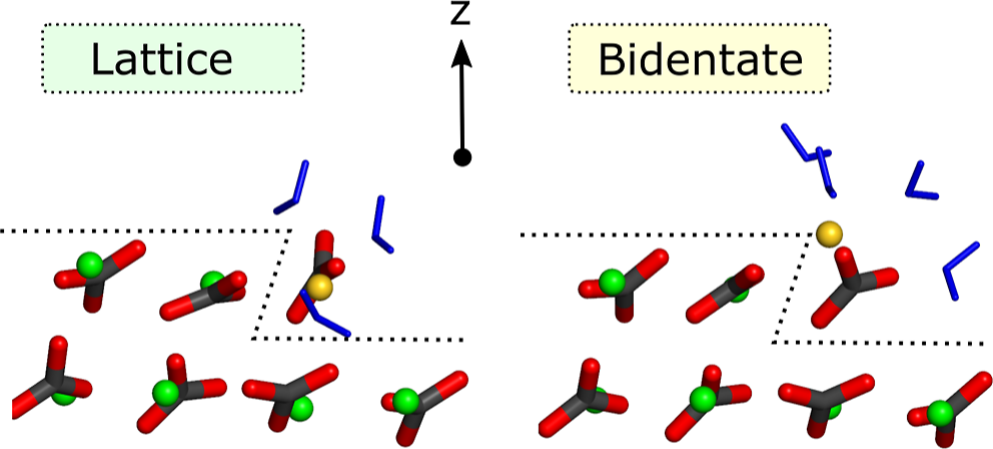
Side views of cross sections of calcite along the step, demonstrating an example of lattice and bidentate configurations. The outline of the steps on which kinks nucleate and propagate are traced with dashed lines. Ca ions are shown in green, C in gray, and O in red. Water molecules are shown in blue and the terminating Ca ion is shown in gold.

The free energy profiles show that the position of the thermodynamic minimum depends on the kink type; some kinks prefer the lattice configuration while others prefer the bidentate configuration (Figure 2). Ca ions prefer to adsorb to the lattice configuration in 6 of the 8 cases. For *a* and *d* kink types, all Ca ions have a thermodynamic minimum at the lattice site. For *b* and *c* kink types, we find a greater variation in the free energy landscapes, where some prefer the bidentate configuration. Where the lattice configuration is preferred, ⟨*N*_c_⟩ corresponds to roughly 3, implying that a total of three coordinated water molecules is the most stable configuration (see Table 1). Where the bidentate configuration is preferred, ⟨*N*_c_⟩ is typically about 4.5, implying that the number of coordinated water molecules fluctuates between 4 and 5. Snapshots of each Ca kink in its most stable configuration are shown in Figure S5.

### 3.2 CO_3_ Adsorption Free Energies

The free energy surfaces for a CO_3_ ion adsorbing to four different Ca-terminated kinks are shown in Figure 1. The complete free energy landscapes are shown in Figure S6. Table 1 summarizes the simulation free energy differences and adsorption free energies. Unlike Ca kinks, of which all eight were studied, we have only shown the results for four kinks. This is because any attempts to study b or c CO_3_ kinks resulted in a water molecule becoming trapped under the kink-terminating Ca ion during the simulation. In this situation, the Ca ion would otherwise transition to its bidentate configuration. However, due to the harmonic tethering of the Ca ion, it was unable to do so. The result was that the simulation configurations became unstable, and metadynamics simulations ran into convergence issues. This issue could be solved by applying a dual adsorption method such as the one discussed in Section 2.3. However, these simulation require a far longer convergence time (∼3 μs) and are therefore beyond the scope of this study.

The free energies show that CO_3_ ions generally adsorb more strongly than Ca ions. The adsorption energies for the CO_3_ adsorbates also show less spread than for the Ca adsorbates, with a total span of 5 kJ/mol between the lowest and highest values, compared to 16 kJ/mol for Ca.

Local free energy minima correspond to both lattice and bidentate configurations. The lattice configuration requires the full dehydration of the Ca-terminated kink site to which the CO_3_ ion binds, while the bidentate configuration does not. Significantly, half of the CO_3_-terminated kink sites prefer to adopt the bidentate configuration. Only the CO_3_ kinks have a preference for the lattice configuration. By contrast, Ca ions mostly preferred the lattice configuration. This difference is likely explained by the water molecules at Ca-terminated kinks, the residence times of which are likely to be far larger than those of lone Ca ions.^28^ The removal of water at kink sites therefore comes at a larger free energy cost than the removal of water at lone ions. The most stable bound configurations of CO_3_ ions are shown in Figure S7.

### 3.3 Example of a Multistep Kink Growth Mechanism

**Figure 3:**
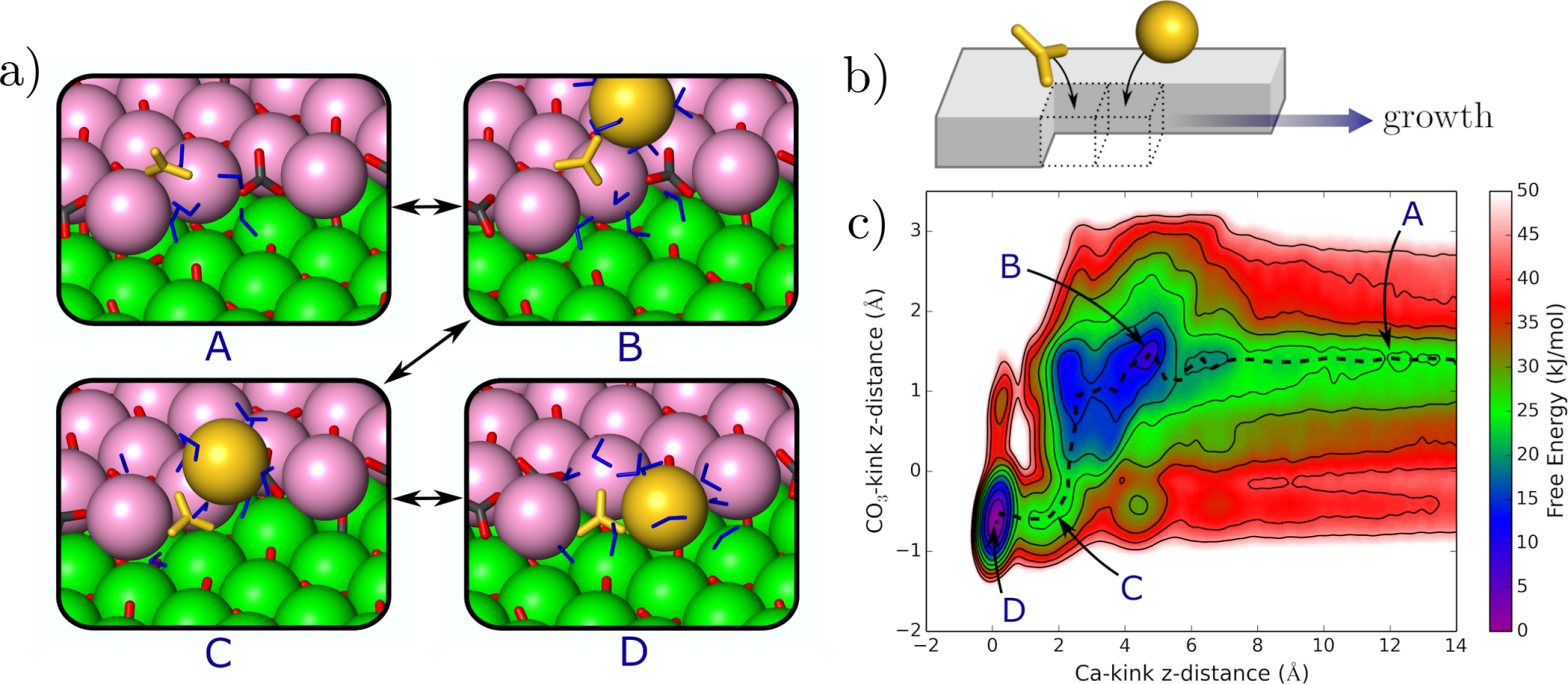
(a) Four snapshots (A–D) illustrate the multistep growth mechanism. Here, Ca atoms in the upper terrace are shown in pink. The two terminating ions are shown in gold. The perspective of the snapshots is one which directly faces the step, which runs horizontally. The kinks grow from the left side. (b) Schematic depicting the perspective of the snapshots and the direction of growth of the kink. (c) Free energy as a function of the position of the CO_3_ and Ca ions adsorbing to the *d*(i) kink. A third reaction coordinate that accounts for dehydration has been integrated out. The minimum free energy pathway is traced with a dashed black line.

Because Ca and CO_3_ ions adsorb preferentially to the bidentate configuration for some of the kinks, it cannot be assumed that kinks always grow via the sequential adsorption of ions directly to the lattice sites. Rather, the adsorption of ions into the lattice sites must involve a more complex process, where ions transition to the lattice site only after the adsorption of a second solute (and possibly more). To demonstrate such a mechanism, we calculated the free energy surface for a CO_3_ ion and a Ca ion adsorbing to a d(i) Ca-terminated kink (Figure 3).

There are four distinct steps to the growth process, labeled A–D in Figure 3. First, the CO_3_ ion adsorbs to the kink in the bidentate configuration (A). The Ca ion then adsorbs to the bidentate CO_3_ ion by sitting approximately 5 Å above the step (B). The CO_3_ ion transitions to the lattice site, pulling the Ca ion into a bidentate configuration (C). This comes at a free energy cost. Finally, the Ca ion transitions into its lattice configuration (D). This completes the process of adsorption, and it is found that D is the most stable configuration. This result is significant as it demonstrates that even the CO_3_ ion with the least stable lattice configuration is stabilized at the lattice configuration through the insertion of one additional ion. We also note that three of the four kink types studied have a preference for the bidentate configuration, while all a and d Ca kinks prefer to adopt their lattice configuration. We therefore expect a similar multistep process to occur for all other a and d CO_3_ kink types. It is worth stressing, however, that the mechanism demonstrated here is only an example of a multistep kink growth mechanism, and that we are not assuming that this result will carry over to other kink types which reside in a bidentate configuration. Nevertheless, the results shown in Figure 2 demonstrate that many terminating ions (4 of the 12 studied) must require one (or more) additional ions to adsorb before a full transition to the lattice site can take place. Ideally, all kink types which prefer to sit in their bidentate configuration should be studied. However, the free energy plot shown in Figure 3 took a total of 3 μs to convergence. Repeating this process for five kink types would require multiple simulations over very large time-scales and is therefore beyond the scope of this paper.

### 3.4 Role of Cation Dehydration in Limiting Kink Growth

Cation dehydration is generally believed to limit the rate of ionic crystal growth,^29−32^ although recent evidence suggests this may not be true for the growth of calcium minerals.^33^ For adsorption into the bidentate configuration, our simulation results broadly support this new perspective: we find that, for all of the kinks that we have sampled, the ions must overcome only a ∼1 *k*_B_*T* barrier to transition from solution to the bidentate configuration. The solutes will therefore initially adsorb to kinks at a rate determined by diffusion rather than by a reaction barrier.

For some kinks, the lattice configuration is more stable than the bidentate configuration, and there typically exists a substantial barrier from the latter to the former. However, because the barrier from bidentate to solution is generally larger than the barrier from bidentate to lattice, the adsorbate is effectively captured by the kink site as soon as it reaches the bidentate configuration.

## 4 Conclusions

Many of the ions that terminate calcite kinks have a tendency to reside in a bidentate configuration, rather than fit directly into the lattice. They sit above the kink, binding to two ions and causing minimal displacement of water molecules. The integration of these ions into the kink lattice site requires the adsorption of an additional ion, and so calcite kinks do not generally grow via a sequence of independent adsorption events as assumed in classical models. This multistep kink propagation process is analogous to what is observed for kink nucleation, in which solutes initially adsorb to the upper terrace before the adsorption of another ion. Future molecular simulation studies of impurities adsorbing to kinks must therefore take into account whether the kink to which an impurity binds resides in its lattice or bidentate configuration.

## Supporting Information Available

Simulation parameters and cell dimensions, discussion on dehydrating Ca adsorbates, discussion on dehydrating Ca-terminated kinks, discussion on calculating free energy surfaces, metadynamics parameters, discussion on calculating adsorption free energies from simulation free energy differences, derivation of formula relating simulation free energy differences and adsorption free energies, schematic of simulation cell detailing cell sizes, free energy plots for Ca adsorbates with dehydration included as a collective variable, comparison of Ca adsorbate free energies with and without dehydration included as a collective variable, all Ca adsorbate free energies as a function of their *z*-distance from the kink site, snapshots from simulations detailing the most stable bound state of each Ca adsorbate, free energy for every CO_3_ adsorbate as a function of its distance from the kink site and the hydration of the kink site, and snapshots from simulations detailing the most stable bound state for each CO_3_ adsorbate (PDF)
