# Supplementary material for: Calcite Kinks Grow via a Multistep Mechanism
Source: J Phys Chem C Nanomater Interfaces. 2022 Sep 13;126(37):15980–5. doi: 10.1021/acs.jpcc.2c04116 (PMC9514807; doi:10.1021/acs.jpcc.2c04116)
Supplement: Supplementary file 1 — jp2c04116_si_001.pdf [file jp2c04116_si_001.pdf]

Supplementary Information: Calcite Kinks Grow Via a Multi-Step Mechanism

Alexander Broad,* Robert Darkins, Dorothy M. Duffy, and Ian J. Ford

London Centre for Nanotechnology, University College London, 17-19 Gordon Street, London, WC1H 0AH, UK

E-mail: a.broad.17@ucl.ac.uk

1 Materials and Methods

1.1 Simulation details

In all simulations, we used a timestep of 1 fs. The temperature of the simulation was kept at 300 K using a Nosé-Hoover algorithm with a relaxation time of 100 fs. When relevant, the same method with a relaxation time of 1000 ps was used to set the pressure to zero. All long-range electrostatics were handled using a PPPM method with an accuracy of 10^{-4}.

The simulation cell consisted of a slab of calcite, periodic in the x- and y-directions, and a ≈4 nm water-filled gap dividing the slab from its periodic images in the z-direction. In all simulations, an elevated terrace was added to the crystal. The elevated step spanned the entire length of the simulation box along the x-direction.

To preserve the crystal structure, the number of repetitions of the calcite unit cell depended on the type of kink exposed. When an a- or d-kink was exposed, the box size corresponded to 9 and 13 repetitions of the unit cell in the x- and y-direction respectively, For b- and c-kinks, the box size corresponded to 11 and 13 repetitions of the unit cell in the x- and y-direction respectively. In the z-direction, the box length was given by 6 repetitions of the calcite unit cell in addition to the 4 nm gap. In order to allow the periodicity of the crystal as well as a single exposed kink site on each step, the angle between x and y cell vectors was set to 93.9°for a- and d-kinks, and 82.8°for b- and c-kinks (90°would correspond to an orthogonal simulation cell). The setup of the simulation cell is depicted in figure S2.

For every kink type studied, simulations consisted of an initial simulation where the z-length of the box was relaxed under zero pressure for about 1 ns. From these simulations, the box lengths were averaged, and simulations were run under NVT using the average z-length obtained from these initial simulations. Every simulation including these NPT simulations consisted of an equilibration process of 100 ps. In every simulation, the position of the calcite slab was kept in place with a series of harmonic tethers acting on a select group of Ca-ions roughly in the centre of the slab.

1.2 Dehydrating Ca adsorbates

We explored the need to explicitly dehydrate the Ca ion that adsorbs to a kink by using the water oxygen coordination number of the Ca ion,

$$\mathrm{CN} = \sum_j \frac{1 - \left(\frac{r_j - d_0}{r_0}\right)^n}{1 - \left(\frac{r_j - d_0}{r_0}\right)^m} \tag{S1}$$

where r_j is the distance between the Ca ion and water oxygen j, and d_0, r_0, n and m are constants. We used the values $d_0 = 2.1$; $r_0 = 1$; $n = 4$; $m = 8$ in accordance with previous studies.[1] Figure S3 shows the free energy surface for two different kinks as a function of the z coordinate of the Ca ion and the coordination number CN. Computing the free energy profile without explicit dehydration produces a similar free energy profile (Figure S4). We conclude that it is not necessary to explicitly drive dehydration of the Ca ion.

1.3 Dehydrating Ca-terminated kinks

Preliminary studies showed that, unlike lone ions or step sites, equation S1 cannot be easily applied to kink Ca ions. Any attempts to dehydrate the kink Ca ion using equation S1 led to major convergence issues. This was because equation S1 was unable to provide a suitable distinction between the two coordinated and uncoordinated states. This issue was not overcome by adjusting any of the parameters in equation S1. Instead, we opt for the nearest distance approximation, as detailed in the main text.

$$\mathrm{ND}_{\text{Ca-O}_\text{w}} = \frac{\beta}{\log \sum_j \exp\left(\frac{\beta}{r_j}\right)} \tag{S2}$$

where r_j is the distance to atom j and β is a parametrisable constant. In all simulations where the binding of CO_3 ions is measured, equation S2 is used to dehydrate kink Ca ions, with β set to 10 nm. Although the issue with equation S2 only exists for acute sites, equation S2 is also applied to CO_3-terminated obtuse sites for consistency.

1.4 Calculating free energy surfaces

In order to effectively explore the free energy landscape around the kink site, we expect that two sets of reaction coordinates are required. First: the position of the additive; second: the hydration of the exposed calcium ion. A comprehensive description of the reaction pathways would require the inclusion of all three coordinates of the solute, as well as the hydration of the relevant Ca ion. However, this would be extremely computationally expensive. We therefore proceed by biasing only the necessary dehydration parameter as well as one spatial coordinate. The coordinate we use is the solute position normal to the {10.4} surface. This coordinate corresponds to the z-coordinate of the solute used in simulations. We define the location of the empty lattice site (the location roughly at which a solute would adsorb) as having (x,y)-coordinates of (0,0). The x- and y-coordinates of the solute (either Ca or C for the case of CO_3) were both constrained to a region spanning between -2 Å and 2 Å, using harmonic walls with a spring constant, k, of 100 kJ/mol/Å. An additional offset, o was added to the harmonic walls such that the harmonic potential is given by $K(x-a+o)^2$ when $x > a$. Here, we set o to 0.5 Å.

1.4.1 Metadynamics parameters

In all metadynamics simulations, Gaussian hills of height k_BT were deposited every ps. The widths of the hills were set to 0.2 Å for the z-coordinates of the ions. For dehydrating, the Gaussian width (σ parameter) was set to 0.1 Å when equation S1 was used, and 0.2 Å when equation S2 was used. The multiple walkers algorithm[2] was applied to 12 parallel simulations communicating every 10 ps. Well tempering[3] was also applied to accelerate convergence. The bias factor was initially set at 30, and was subsequently adjusted during simulations. Simulation times varied between approximately 300 ns and 3 μs depending on the volume of reaction coordinate space required.

1.5 Adsorption free energies

As discussed in the main text, adsorption free energies are distinctly different from simulation free energy differences, and that the difference between the two reduces to a set of entropy corrections. By assuming that dissolved solutes behave as an ideal gas, the adsorption free energy, ΔG_{ads}, may be given in terms of the simulation free energy difference, ΔG_{sim} as follows.

$$\Delta G_{\text{ads}} = \Delta G_{\text{sim}} + T\Delta S_{\text{ref}} \tag{S3}$$

where T is the system temperature and ΔS_{ref} is a reference entropy that represent the the relative configurational freedoms of adsorbed and dissolved states. ΔS_{ref} is given by:

$$T\Delta S_{\text{ref}} = k_B T \log \left[\frac{V_{1mol}}{l_x l_y \int_a^b \exp\left(-\frac{G(z)-G_{min}}{k_B T}\right) dz} \right] \tag{S4}$$

Here, a and b are the lower and upper limits on the z-values which can be associated with adsorbed states. Their exact values are not especially important, as long as they fully encompass the free energy well associated with adsorption. G_{min} is the smallest value of the free energy at the base of the well. V_{1mol} is the average volume occupied by an impurity at a concentration of 1 mol (1660.5 Å^3), and l_x and l_y are the spacings between the lower and upper walls that constrain the positions of the additive in the x- and y-directions respectively. In this case, both l_x and l_y equal 6. A full derivation for the above equations can be found below in section 2.

2 Derivations

2.1 Adsorption free energy from simulation free energy difference

The adsorption free energy, ΔG_{ads}, is given by

$$\Delta G_{\text{ads}} = -k_B T \log\left(\frac{P_{\text{ads}}}{P_{\text{diss}}}\right) \tag{S5}$$

where P_{ads} and P_{diss} are the probabilities of finding the impurity in an adsorbed or dissolved state respectively at an activity of 1 mol. In order to derive ΔG_{ads}, from simulations, first consider the probability of adsorption in the context of the simulation. Consider the example free energy surface in figure S1. The solute can be considered adsorbed between the regions a and b. Between b and c, the solute is not fully adsorbed, but is still somewhat associated with the surface. When the free energy surface becomes flat, i.e. beyond c, the solute is fully dissolved. The probability of finding the adsorbate between points a and b, P_{a-b} (adsorbed), divided by the probability of finding the adsorbate between points c and d (dissolved), P_{c-d}, is given by

$$\frac{P_{a-b}}{P_{c-d}} = \frac{\int_a^b \exp\left(-\frac{G(z)}{k_B T}\right) dz}{\int_c^d \exp\left(-\frac{G(z)}{k_B T}\right) dz} \tag{S6}$$

Since $G(z)$ should be flat between regions c and d, we can define $G(z)$ between c and d to be $G(z) = G_{\text{min}} - \Delta G_{\text{sim}}$, where G_{min} is the minimum value of $G(z)$ and ΔG_{sim} is the simulation free energy difference (see figure S1). Note that ΔG_{sim} is negative, hence the minus sign. The ratio of probabilities therefore simplifies to

$$\frac{P_{a-b}}{P_{c-d}} = \frac{\exp\left(-\frac{\Delta G_{\text{sim}}}{k_B T}\right)}{(d-c)} \int_a^b \exp\left(-\frac{G(z) - G_{\text{min}}}{k_B T}\right) dz \tag{S7}$$

We now consider the true probabilities of finding asp in an adsorbed or dissolved state. The probability of the impurity being adsorbed is entirely determined by the probability of finding the impurity between a and b. Assuming the dissolved impurity behaves as an ideal

gas, the probability of finding the impurity in a dissolved state is proportional to the average volume it occupies in solution at a concentration of 1 mol, $V_{1\mathrm{M}}$. This probability can be related to P_{c-d} by multiplying by the ratio of accessible volumes i.e. $V_{1\mathrm{M}}/l_x l_y(d-c)$. We therefore have:

$$\begin{aligned} P_{\mathrm{ads}} &\propto P_{a-b} \\ P_{\mathrm{diss}} &\propto P_{c-d}\frac{V_{1\mathrm{M}}}{l_x l_y(d-c)} \end{aligned} \tag{S8}$$

which simplifies to

$$\frac{P_{\mathrm{ads}}}{P_{\mathrm{diss}}} = \frac{P_{a-b}}{P_{c-d}}\frac{l_x l_y(d-c)}{V_{1\mathrm{M}}} \tag{S9}$$

It should be noted that the accessible volume in the simulation is not exactly equal to $l_x l_y(d-c)$, as the solute it still able to explore regions beyond the harmonic walls used to constrain the x- and y-positions of the particle within the column. However, the high spring constant used, as well as the use of offsets (see section 1.4) minimises this contribution to the accessible volume. We calculate that, ultimately, the regions beyond the harmonic walls reduce the adsorption free energy by 0.05 kJ/mol. Since this value is much smaller than the error of the simulation, and has no effect on the output of the results, we do not need to include it in calculations.

Equation S9 can finally be combined with equations S5 and S7 to give

$$\Delta G_{\mathrm{ads}} = -k_B T \log\left(\frac{P_{\mathrm{ads}}}{P_{\mathrm{diss}}}\right) = \Delta G_{\mathrm{sim}} + k_B T \log\left[\frac{V_{1\mathrm{M}}}{l_x l_y \int_a^b \exp\left(-\frac{G(z)-G_{min}}{k_B T}\right) dz}\right] \tag{S10}$$

Note that the result is no longer dependent on c and d.

3 Figures

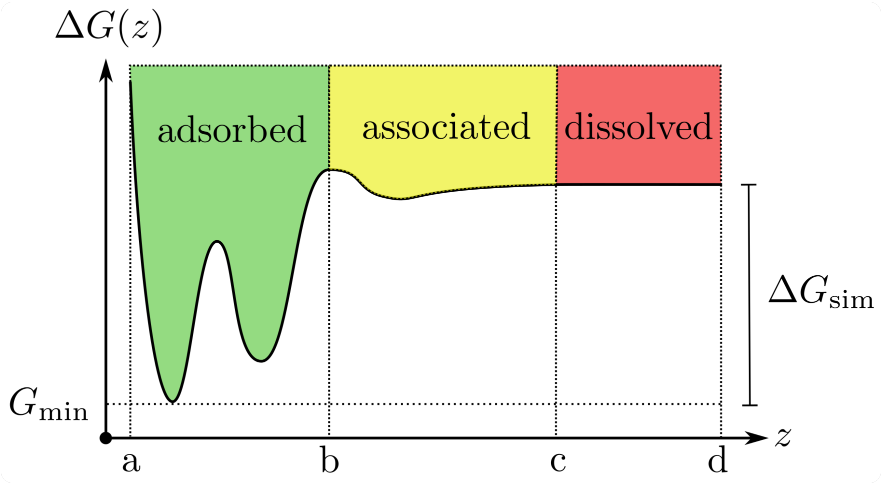

Figure S1: Example of a free energy surface in which ΔG_{sim}, G_{min}, a, b, c and d are defined. Note that this example does not represent the shape of every free energy surface in this paper, but the theory equally applies in other settings, as long as the free energy surface is flat between c and d. It should also be noted that the exact positions of a and b are not too important, as the integral over the probability density will be dominated by the regions near the thermodynamic minimum.

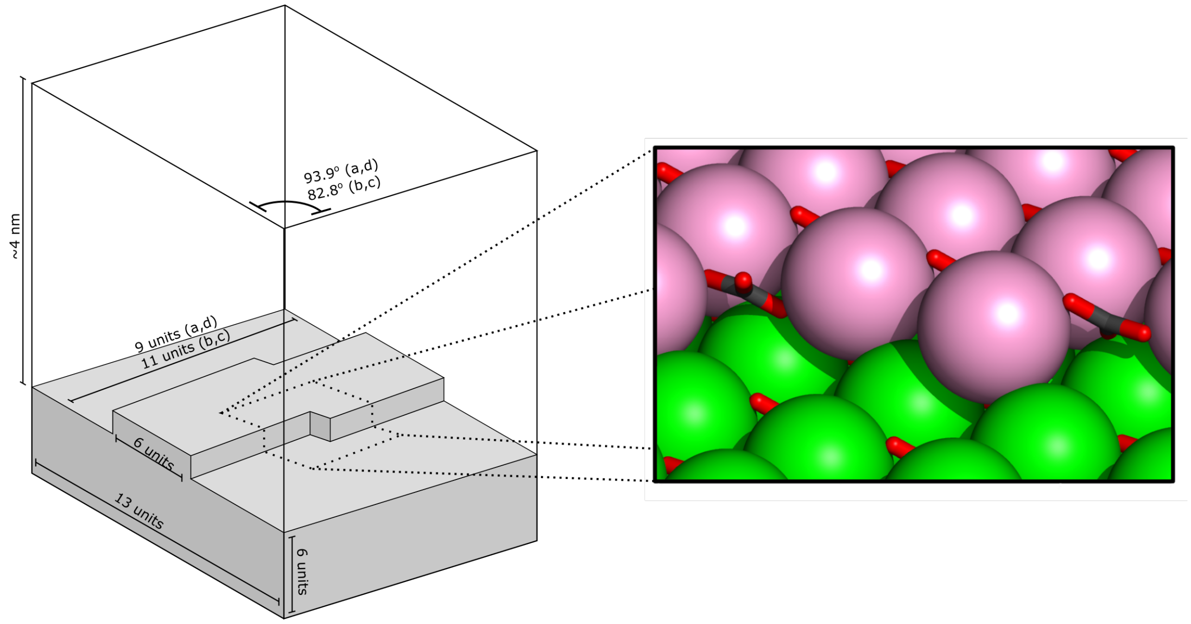

Figure S2: Schematic of the simulation cell setup designed to isolate a particular kink site in simulations. The cell dimensions are also labelled. For calcite, calcium is depicted in green for the bulk crystal and pink for the upper terrace, carbon is depicted in grey, and oxygen in red.

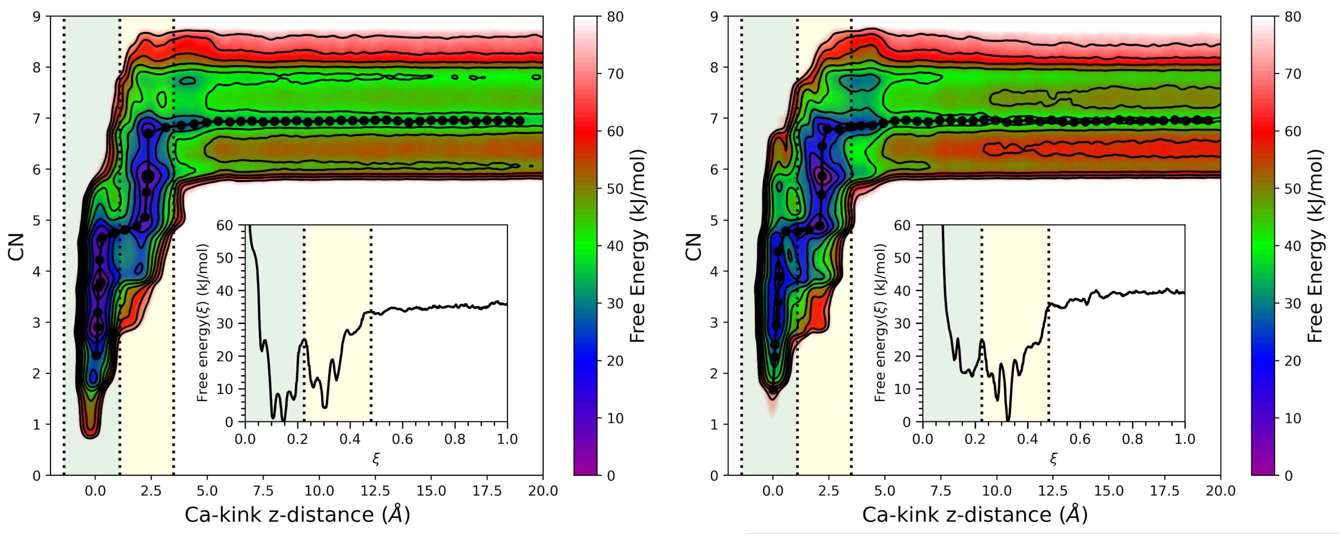

Figure S3: Ca-kink interaction free energy as a function of the Ca-kink distance and Ca-O_w coordination number for the a(ii) kink (left) and the c(i) kink (right). The dotted beads show the MFEP for Ca attachment to the kink site. The inset graph shows the value of the MFEP as a function of the dimensionless parameter that maps it.

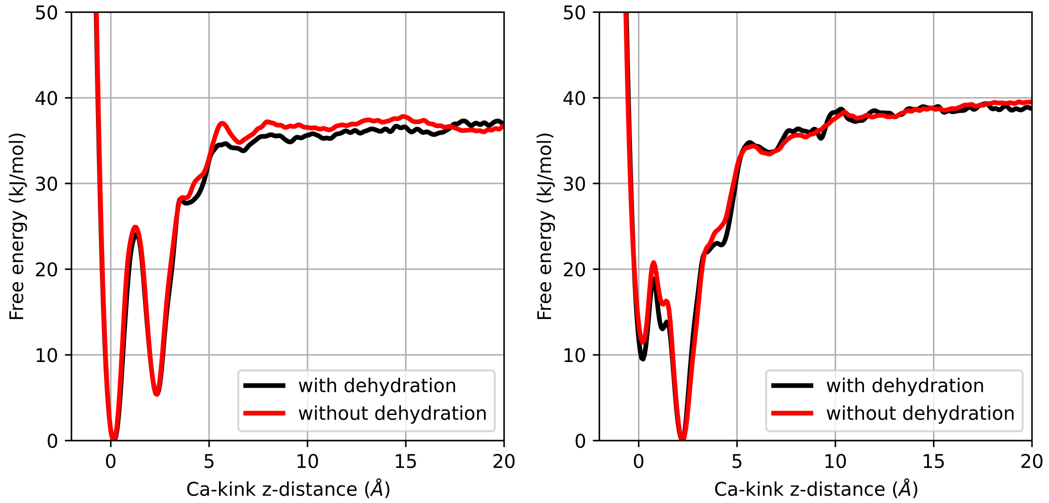

Figure S4: Free energy surfaces for Ca ions as a function of their distance from their respective kink sites for the a(ii) (left) and c(i) (right) kinks. A good agreement is found between simulations where dehydration is included as a reaction coordinate, and when it isn't.

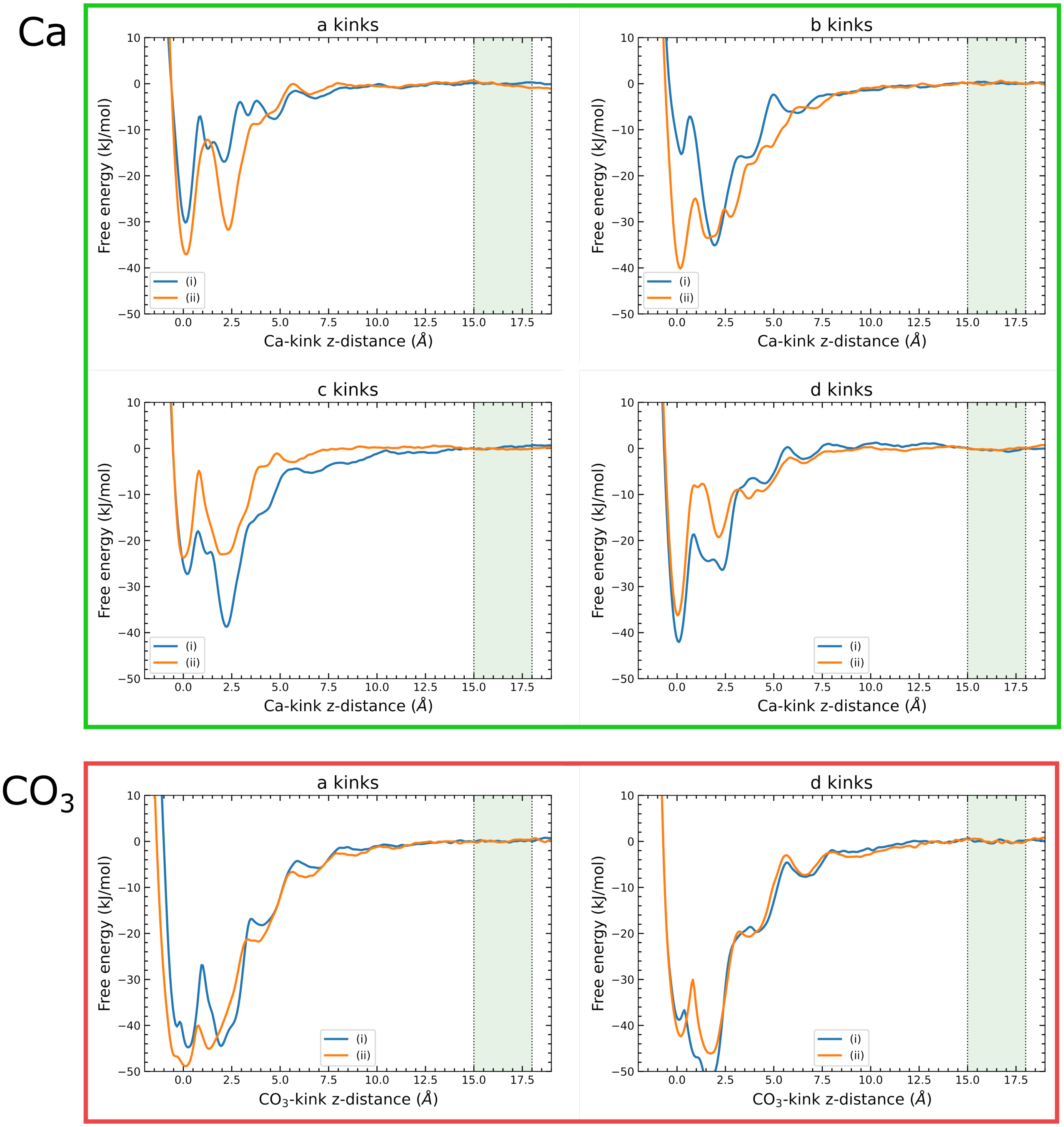

Figure S5: Free energies as shown in figure 1, but with a larger region shown. ΔG_{sim} is calculated by computing the difference between the minimum free energy value, and the free energy averaged over the green region. The free energies here are normalised to the latter value (as are the ones shown in figure 1).

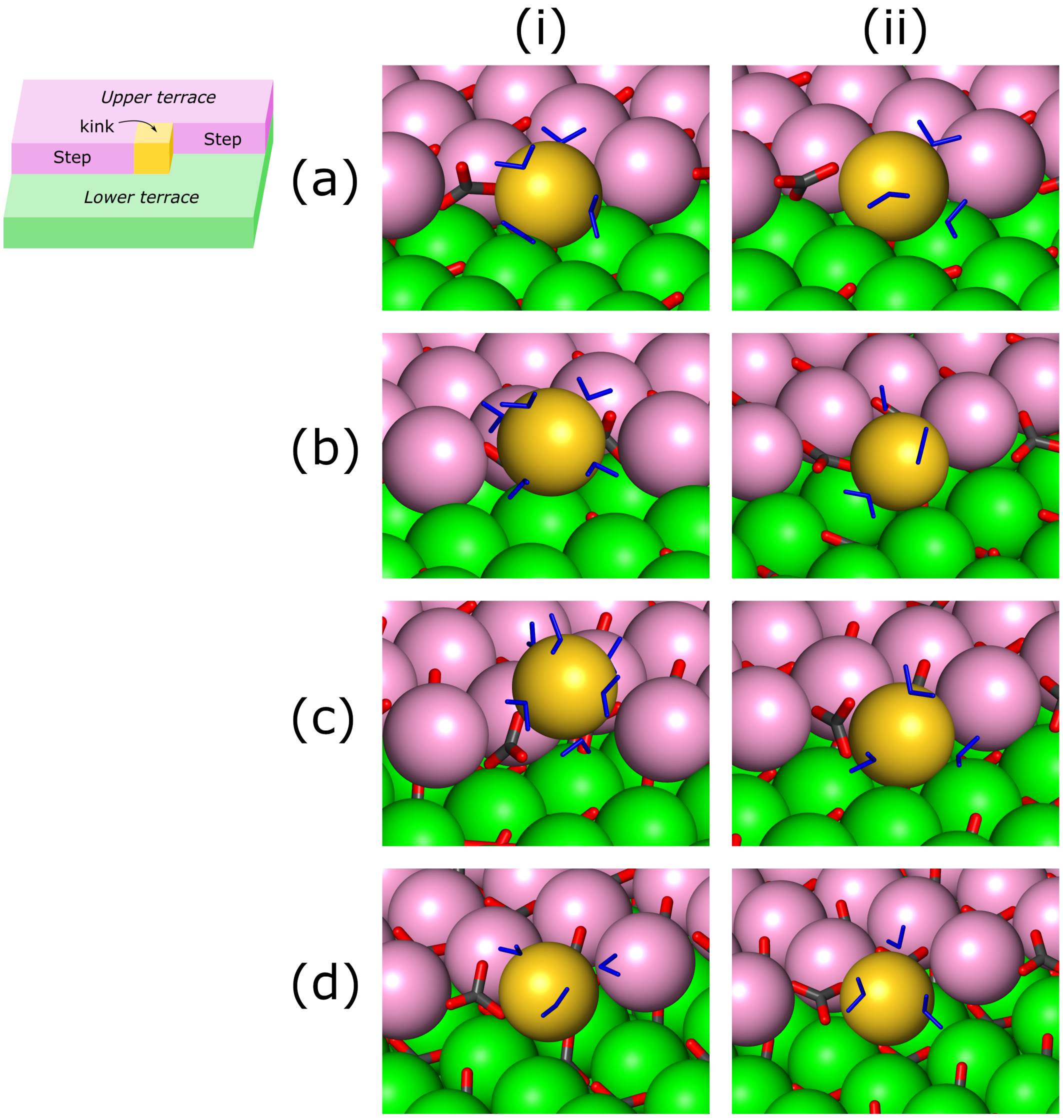

Figure S6: snapshots from simulations showing the strongest binding configurations for every Ca terminated kink. The water molecules coordinated with the Ca kink are also shown. The colouring of the atoms corresponds to that of figure S2. The terminating CO_3 ions are shown in gold, and the water molecules are shown in blue.

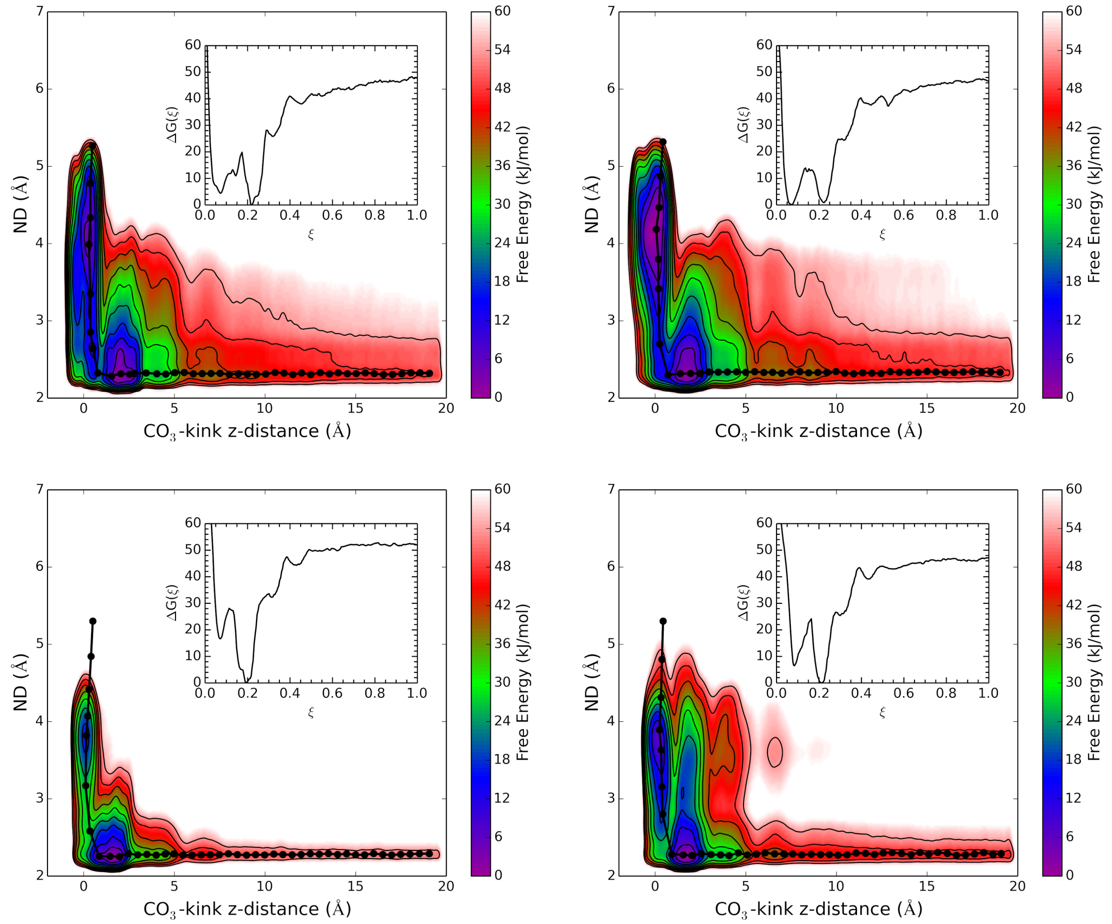

Figure S7: Free energy surfaces for CO_3-terminated kinks as a function of CO_3-kink z-distance and ND (see equation S2) for a(i) (top-left), a(ii) (top-right), d(i) (bottom-left), and d(ii) (bottom-right) kinks. The connected black dots trace the MFEP, which is also shown in the insets.

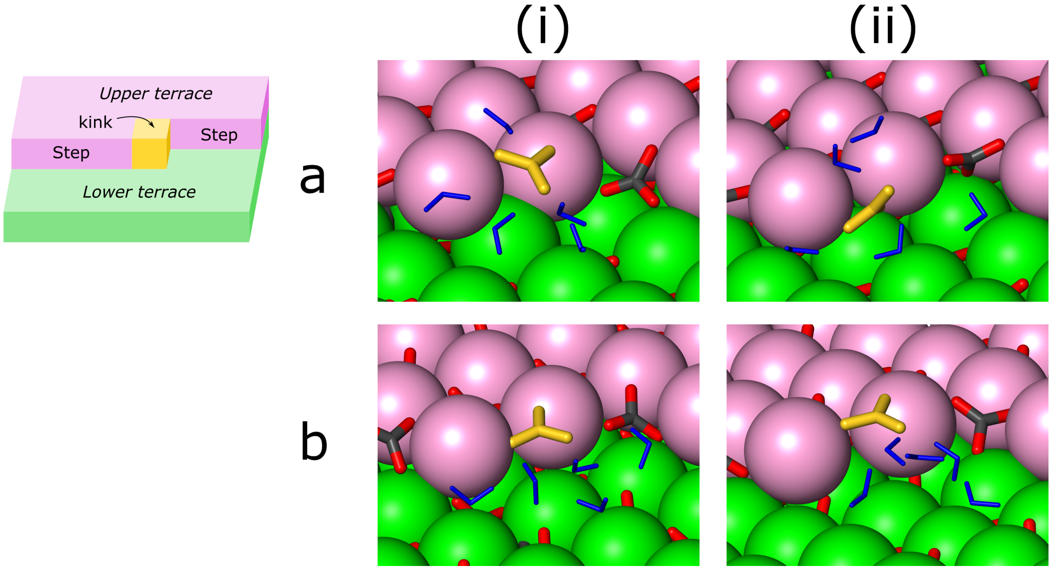

Figure S8: snapshots from simulations showing the strongest binding configurations for every CO_3 terminated kink. The nearest five water molecules to the kink site are also shown. The colouring of the atoms corresponds to that of figure S2. The terminating CO_3 ions are shown in gold and the water molecules in blue.

References

(1) Raiteri, P.; Demichelis, R.; Gale, J. D. Thermodynamically consistent force field for molecular dynamics simulations of alkaline-earth carbonates and their aqueous speciation. *The Journal of Physical Chemistry C* **2015**, *119*, 24447–24458.

(2) Raiteri, P.; Laio, A.; Gervasio, F. L.; Micheletti, C.; Parrinello, M. Efficient reconstruction of complex free energy landscapes by multiple walkers metadynamics. *The journal of physical chemistry B* **2006**, *110*, 3533–3539.

(3) Barducci, A.; Bussi, G.; Parrinello, M. Well-tempered metadynamics: a smoothly converging and tunable free-energy method. *Physical review letters* **2008**, *100*, 020603.
